# School-based interventions for promoting food and nutrition literacy (FNLIT) in elementary school children: a systematic review protocol

**DOI:** 10.1186/s13643-020-01339-0

**Published:** 2020-04-22

**Authors:** Azam Doustmohammadian, Nasrin Omidvar, Elham Shakibazadeh

**Affiliations:** 1 Department of Nutrition Research, National Nutrition and Food Technology Research Institute (WHO Collaborating Center), Tehran, Iran; 2 Faculty of Nutrition Sciences and Food Technology, Shahid Beheshti University of Medical Sciences, Tehran, Iran; 3 Department of Community Nutrition, National Nutrition and Food Technology Research Institute (WHO Collaborating Center), Tehran, Iran; 4 Department of Health Education and Promotion, School of Public Health, Tehran University of Medical Sciences, Tehran, Iran

## Abstract

**Background:**

Food and nutrition literacy is a newly emerged concept to connect food-related knowledge and skills to healthy diets. Its promotion is important to protect children as they eat too many low-nutrient, high-energy foods*.* Food and nutrition literacy promotion needs multi-dimensional interventions. In the process of developing an intervention to promote food and nutrition literacy, the present review protocol aims to critically examine the evidence in the area of school-based interventions for promoting food and nutrition literacy (FNLIT) in elementary school children.

**Methods:**

We will search PubMed/MEDLINE, EMBASE, Web of Science, CENTRAL, and ProQuest (from inception onwards). Additional studies will be identified through manual searching of reference lists. Quantitative studies (e.g., randomized controlled trial, quasi-randomized trials, and cluster randomized trials) evaluating the effect of interventions to promote food and nutrition literacy in elementary school children (5–12 years old) will be included. Main outcomes will be food and nutrition literacy at functional, interactive, and critical levels. Secondary outcomes will be dietary intake indicators (e.g., healthy eating index, DDS) and health outcome measures (e.g., reduction in BMI and less weight gain). Two reviewers will independently screen all citations, full-text articles, and abstract data. Potential conflicts will be resolved through discussion. The study methodological quality (or bias) will be appraised using appropriate tools. If feasible, we will conduct random effects meta-analysis. The quality of the included studies will separately evaluate using the validated Quality Assessment Tool for Quantitative Studies, developed by the Effective Public Health Practice Project (EPHPP). Data will be extracted by two reviewers from the identified relevant literature. Standard data synthesis and analysis will be used for the review.

**Discussion:**

This systematic review will summarize the evidence regarding the components, implementation methods, and effectiveness of the interventions of food and nutrition literacy promotion in elementary school children. The results of this review will provide a useful reference for policymakers and curriculum developers to assess education curricula and develop practical learning and teaching strategies to improve students’ food and nutrition literacy.

**Systematic review registration:**

PROSPERO (CRD42019135118)

## Background

Non-communicable diseases (NCDs) are the major cause of mortality and morbidity globally [1]. The World Health Organization (WHO) has estimated that by 2020, NCDs will account for 80% of the global burden of disease [2]. In developing countries, including Iran, the prevalence of NCDs has increased over the past few years [3]. Most NCDs are associated with modifiable lifestyle factors [4]. It is well documented that high-risk behaviors such as meal skipping, unhealthy eating behaviors, and low physical activity is increasing among children and adolescents [5–8]. This has resulted in growing prevalence of overweight and obesity among children and adolescents in many countries [9, 10].

Health literacy is considered as one of the most important personal skills to enable individuals to control health determinants. Nutbeam described health literacy as “the cognitive and social skills which determine the motivation and ability of individuals to gain access to, understand and use information in ways which promote and maintain good health” [11]. He highlighted the importance of achieving health literacy at functional, interactive, and critical levels [11]. Due to the wide scope of health issues, studies suggest that one should consider health literacy more specifically [12]. As a result, specific areas of health literacy, including food literacy and nutrition literacy, have been proposed and conceptualized. Studies that assessed the relationship between two concepts suggested a wide multifaceted topic that can be called food and nutrition literacy (FNLIT) [13, 14].

Evidence shows that promoting FNLIT is a key factor in healthy food choice and following healthy eating patterns in children and adolescents [15–17]. Increasing evidence show that interventions to improve food and nutrition literacy can have a positive effect on the food and nutrition skills and dietary patterns including food selection, food preparation, increased fruit and vegetable consumption, increased self-efficacy in these areas, and improved diet quality [18, 19].

Guided by Nutbeam’s hierarchical model of health literacy [11], food literacy/nutrition literacy have been categorized in three levels: (1) functional food/nutrition literacy is the knowledge and ability to obtain information from various sources and understanding and comprehending it as well as practical skills and strategies to promote health; (2) interactive food/nutrition literacy includes interpersonal skills with experts, peers, and other people to share and discuss necessary food and nutritional information; and (3) critical nutrition literacy refers to the ability to critically analyze food and nutrition information, understanding the food impact on the environmental as well as having the will to participate in actions to address barriers to nutritional health [11, 20–23].

Functional literacy is considered as the lowest level and critical literacy as the highest [11, 20]. In a study conducted in Iran, the cognitive and skills domains of FNLIT were identified for elementary school children. The cognitive domain included two subdomains, namely, understanding food and nutrition information, and nutritional health knowledge. The skills domain included seven subdomains examining functional FNLIT, interactive FNLIT, food choice literacy, critical FNLIT, and food label literacy. In this study, for the first time, components of FNLIT dimension were identified in elementary school children [14].

Due to the novelty of the concept of FNLIT, a comprehensive intervention to promote FNLIT, especially at the interactive and critical levels, has not been yet developed. Some of the interventions related to the promotion of food literacy/nutrition literacy mentioned in some studies are as follows [15, 24–26]:

- Educating individuals about portion sizes and daily food group, describing and learning how to read nutritional labels and apply them to one’s dietary change by training and video programs
- Training in gardening and planting
- Cookbooks that feature healthy recipes
- Functional guideline for food purchasing using an empty grocery list in accordance with the traffic light-coded food labels
- Media literacy/critiquing a food commercial

The promotion of FNLIT in children seems to be one of the most important elements in improvement of healthy dietary pattern and prevention of diet-related non-communicable diseases for several reasons. First, FNLIT is related to everyday living activities, i.e., food related activities, including eating [27]. Second, it “empowers individuals, households, and communities to protect diet quality through change and strengthen dietary resilience over time” [28]. Children who develop interactive and critical skills of FNLIT find it easier to manage their food choices, resolve conflict of their different interests, interact nutritional information with others, and participate in action to address barriers.

Third, based on the evidence, interventions in the early years of life are recommended to take advantage of the child learning ability and more possibility of successful stabilization of healthy behavior into adulthood [29]. The fourth rationale for the importance of FNLIT promotion is derived from its relevance in the context of school. Schools have direct contact with students for approximately 6 h each day and for up to 13 critical years of their social, psychological, physical, and intellectual development [30]. The school settings have been identified by the WHO as an ideal setting to teach children and adolescents about healthy dietary habits and help them to make healthy and informed food choices [31]. However, lack of documented policies and programs in the field of FNLIT is a problem, especially in developing countries.

According to the studies, FNLIT has multi-dimensional political, cultural, and social characteristics. This multi-dimensional nature of this concept confirms the need for multi-dimensional interventions to improve FNLIT [12, 28]. The first step to develop this type of intervention involves referring to the evidence and modeling based on the effective and successful interventions [32]. This approach guides researchers to find ways to integrate components of prevention programs in ways that are acceptable and meaningful to the school setting and to evaluate results. Therefore, this review aims to collect interventional studies related to FNLIT promotion among school-age children through searching for relevant scientific literature. After the final selection and extraction of the data, components, implementation methods, and effectiveness of the interventions will be identified and reported in the form of a systematic review to be used as a guide in developing interventions with the same purpose.

## Methods

The objectives of this study will be to systematically determine components within interventions that have an impact on children’s food and nutrition literacy (at functional, interactive, and critical levels) among elementary school children (5–12 years old).

The objectives of this study are to:

1. Determine components within interventions that have an impact on food and nutrition literacy (at functional, interactive, and critical dimensions) among elementary school children (5–12 years old).
2. Determine the implementation methods of food and nutrition literacy interventions (at functional, interactive, and critical dimensions) among elementary school children (5–12 years old).
3. Determine the effectiveness of interventions in promotion of FNLIT (at functional, interactive, and critical dimensions) among elementary school children (5–12 years old).

### Study design

The present protocol has been registered within the PROSPERO database (registration number CRD42019135118) and is being reported in accordance with the reporting guidance provided in the Preferred Reporting Items for Systematic Reviews and Meta-Analyses Protocols (PRISMA-P) statement [33] (see checklist in Additional file 1). The proposed systematic review and meta-analysis will be conducted following the guidance in the Cochrane Collaboration Handbook of Systematic Reviews [34]. The methods and results will be reported in accordance with the reporting guidance provided in the Preferred Reporting Items for Systematic Reviews and Meta-Analyses (PRISMA) statement [35].

### Criteria for considering studies for this review

#### Types of studies

Quantitative studies (e.g., case-control studies, pre-post studies, randomized controlled trial, quasi-randomized trials, and cluster randomized trials) evaluating the effect of interventions to promote food and nutrition literacy in elementary school children (5–12 years old) will be included.

#### Types of participants

We will assess all studies whose target group are elementary school students (5–12 years old).

#### Types of interventions

Any type of studies available in English featuring interventions that contain one or more components of food literacy/nutrition literacy [36], including understanding of food and nutrition information, knowledge of food and nutrition, the skill of finding information from various sources, meal and snack preparation, increasing the consumption of fruits and vegetables, reducing the consumption of prepared foods, healthy food choice, and the skill of reading and analyzing food labels in school setting, will be included in the review without considering the time limit. The studies that focus on nutritional interventions related to diseases such as type II diabetes and obesity will be excluded.

#### Types of outcome measures

Referring to Nutbeam’s tripartite model [11, 21], which considers three levels of literacy (functional, interactive, and critical), the main outcomes evaluated in this review consist of food and nutrition literacy at functional, interactive, and critical levels. Examples of related behaviors at each level are as follows:

Functional food and nutrition literacy: improve meal planning, prioritizing healthy meal choices, reading nutrition facts labels, cooking skill confidence, and desire to fewer fast food meals. Increase in fruit and vegetable and whole grain consumption [37] and preparing fruits or vegetables in a new way [38].

Interactive food and nutrition literacy: family-child feeding interactions [37], skill of saying “no” to unhealthy food, and emotional skills [14].

Critical food and nutrition literacy: trying ethnic and unfamiliar food [39], food label literacy [40], improving school social environment, increasing school community connections [41], and engagement with issues of social justice and equity in food systems [42].

Successful interventions and those that include theories and hands-on activities to enhance literacy will be taken into account.

Secondary outcomes include improvement in diet quality (e.g., healthy eating index) [16], dietary intake indicators (e.g., DDS), reduction in BMI and less weight gain [43], and indicators of quality of life/wellbeing [44].

The study outcomes will not be a criterion to enter the study, and all the positive and negative outcomes will be checked.

### Search methods for identification of studies

#### Electronic searches

The primary source of literature will be a structured search of major electronic databases (from inception onwards): PubMed/MEDLINE, EMBASE, Scopus, Web of Science, and CENTRAL. The secondary source of potentially relevant material will be a search of the grey or difficult to locate literature, including ProQuest and Google Scholar. We will perform hand-searching of the reference lists of included studies, relevant reviews, or other relevant documents. The literature searches will be designed and conducted by the review team which includes two experienced health information specialists. The search will include a broad range of terms and keywords related to “food literacy,” “nutrition literacy,” “children,” “adolescents,” and “interventions”. A draft search strategy for multiple databases is provided in Additional file 2.

### Data collection and analysis

#### Selection of studies

All the identified studies of different sources will be transferred to Endnote and systematically de-duplicated, and a merged library will be created. Two reviewers will independently screen the titles and abstracts according to a pre-defined inclusion criteria checklist and will exclude unrelated ones. In case of disagreement between the reviewers, the judgment of article inclusion in the study will be made by a third person. The full texts will be read by the two individuals separately, and the final decisions will be made based on the checklist of inclusion criteria. The PRISMA (Preferred Reporting Items for Systematic Review and Meta-Analyses) flowchart [33] will be used to document the selection process.

#### Data extraction and management

A data extraction form will be designed and used to extract information from each study report. Information of interest will include the following:

- Study characteristics: study design, year of publication, journal, year (or period) of study conduct, and geographical location of study conduct
- Participant characteristics: sample size, age (e.g., mean with standard deviation, range), and gender
- Intervention characteristics: intervention (name and type), intervention description (components of intervention, intervention duration/follow-up), and timing of post-intervention evaluation
- Outcome results: data collection method (e.g., validated tools), main outcomes including improvement in FNLIT at functional, interactive, and critical literacy and secondary outcomes including improvement in diet quality (e.g., healthy eating index) [16], dietary intake indicators (e.g., DDS), reduction in BMI and less weight gain [43], and indicators of quality of life/wellbeing [44]

The content of each included studies will be extracted by two team members, independently, and potential conflicts will be resolved through discussion. Authors of primary publications will be contacted for data clarifications or missing outcome data, as necessary.

### Assessment of risk of bias in included studies

#### Appraisal of study quality

Two reviewers will separately evaluate the quality of the included studies using the validated Quality Assessment Tool for Quantitative Studies, developed by the Effective Public Health Practice Project (EPHPP) [45]. This tool was developed in order to provide high-quality systematic reviews of articles relating to public health topics [46]. Eight aspects of quality are assessed: (1) selection bias, (2) study design, (3) confounders, (4) blinding, (5) data collection methods, (6) withdrawals and dropouts, (7) intervention integrity, and (8) analysis, leading to an overall methodological rating of strong, moderate, or weak [46]. The quality of all the included studies will be assessed by the first author. The second and third authors will each check one third of the publications for completeness and accuracy of the quality assessment. Differences in the quality assessment will be resolved by discussion among all of the authors.

#### Data synthesis

The data from each paper (e.g., study characteristics, context, participants, outcomes, and findings) will be used to build evidence tables of an overall description of included studies. If feasible and appropriate, data points from primary observational studies will be used to perform random effects meta-analyses. Since heterogeneity is expected a priori, we will estimate summary estimates (e.g., mean differences, standardized mean difference) and its 95% confidence interval using the random effects model. The random effects model assumes the study prevalence estimates follow a normal distribution, considering both within- and between-study variations. Forest plots will be used to visualize the extent of heterogeneity among studies. We will quantify statistical heterogeneity by estimating the variance between studies using *I*^2^ statistic. The *I*^2^ is the proportion of variation in prevalence estimates that is due to genuine variation in prevalence rather than sampling (random) error. *I*^2^ ranges between 0 and 100% (with values of 0–25% and 75–100% taken to indicate low and considerable heterogeneity, respectively). We will also report Tau2 and Cochran *Q* test with a *P* value of < 0.05 being considered as statistically significant (heterogeneity). If quantitative synthesis is not appropriate, the findings will be summarized and discussed. Conclusions will be also formed on the basis of the power of each of the studies. After summarizing the results and providing conclusions that lead to improving interventional decision-making, the most successful and effective interventions will be identified**.**

#### Additional analyses

If sufficient studies are identified and data points are available, potential sources of heterogeneity will be investigated further by subgroup or meta-regression analysis according to baseline characteristics and methodological covariates. We plan to conduct analyses by sex (girls vs boys) [47], age (e.g., children vs adolescent, midpoint of age range as continuous variable) [36, 47], type of study (RCT, case-control, pre-post, etc.), food literacy/nutrition literacy level (e.g., low, moderate or high), sample size (e.g., < 1000, 1000–5000, or > 5000 participants), and study quality (e.g., low/moderate vs high-risk of bias). Small study effects (or publication bias) will be assessed by inspection of the funnel plots for asymmetry and with Egger’s test [48] and Begg’s test [49], with the results considered to indicate potential small study effects when *P* values are < 0.10.

## Discussion

The combined impact of poor diet and being overweight and obesity rates continue to raise health and economic concerns around the globe. Dietary behavior change and obesity prevention interventions targeting at children are necessary in order to prevent the onset of chronic diseases later in life. Reducing current nutrition-related chronic diseases, such as childhood epidemic of obesity, requires improving their food and nutrition literacy [50]. A goal of food literacy is to bridge the gap between what children know and want to practice. As regards food skills needed for translating knowledge into practice, food and nutrition literacy can have a key role in fostering healthy eating behavior [51].

This systematic review will summarize the evidence regarding the components, implementation methods, and effectiveness of the interventions of food and nutrition literacy promotion in elementary school children. One potential by-product of this review is providing a useful reference for policymakers and curriculum developers to assess education curricula and develop practical learning and teaching strategies to improve students’ food and nutrition literacy. This review will provide insight on the extent to which health providers, educators, families, and students may have an important role in children’s healthy lifestyle behaviors and how their involvement in food and nutrition literacy promotion may contribute to healthier children.

There are several limitations of our planned systematic review methods.

The potential limitations at evidence source (study level) include the following:

- Lack of studies that have assessed the effect of food literacy or nutrition literacy interventions on functional, interactive, and critical dimensions separately.
- Lack of an adequate control or comparator group in studies, limiting the ability to determine the true effect(s) of the intervention.

Since many studies do not directly use the term food literacy or nutritional literacy, we may miss some interventions related to the improvement of some components of food and nutrition literacy. However, the search strategy in our study is highly sensitive to overcome this limitation.

The results of the review will be utilized to assess education curricula and develop practical learning and teaching strategies to improve students’ food and nutrition literacy in Iran which can be utilized by other countries as well.

This review is intended for publication in a peer-reviewed journal. Analyses and scripts will be made publicly available. Any changes to the protocol will be documented as well.

## Supplementary information

**Additional file 1.** PRISMA-P 2015 Checklist.

**Additional file 2: Table S1.** Electronic search strategies.

## Data Availability

Data sharing is not applicable to this article as no datasets were generated or analyzed during the current study.

## Abbreviations

FNLIT: Food and nutrition literacy
WHO: World Health Organization
PRISMA-P: Preferred Reporting Items for Systematic Review and Meta-Analysis Statement-Protocol Extension
RCT: Randomized controlled trial
DDS: Dietary diversity score
BMI: Body mass index
EPHPP: Effective Public Health Practice Project
SMD: Standard mean difference
MD: Mean difference
