# Supplementary material for: School-based interventions for promoting food and nutrition literacy (FNLIT) in elementary school children: a systematic review protocol
Source: Syst Rev. 2020 Apr 22;9:87. doi: 10.1186/s13643-020-01339-0 (PMC7178560; doi:10.1186/s13643-020-01339-0)
Supplement: Supplementary file 2 — Additional file 2: Table S1. Electronic search strategies. [file 13643_2020_1339_MOESM2_ESM.docx]

**Table1. Electronic search strategies**

| **Database** | **Search terms** |
| --- | --- |
| **CENTRAL** | 1. [Food literacy] 2. [Nutrition literacy] 3. [Food skills] 4. [Food preparation] 5. [Garden] 6. [Cooking program] 7. [Selection/Purchase of food] 8. [Food choice] 9. [Consumption of food] 10. [Improvements in fruit and vegetable consumption] 11. [Food budgeting] 12. [Confidence to perform this food work] 13. [Confidence in cooking] 14. [Frequency of using basic ingredients for the preparation of meals] 15. [Confidence in cooking] 16. [Buying less convenience food] 17. [Preparation of meal and snack] 18. [Increased likelihood to taste and experiment with new food] 19. [Increased awareness of food preparation and Production] 20. MeSH descriptor: [Health Literacy] this term only 21. [Nutrition Knowledge, Attitudes, Practice] 22. literacy or literate:ti,ab,kw 23. {or #1-#22} 24. Child* or adolesc* or youth or teenag* or student:ti,ab,kw 25. Intervent*:ti 26. {and #23, #24, #25} |
| **MEDLINE** | 1. Food literacy/ 2. Nutrition literacy/ 3. Food skills/ 4. Food preparation/ 5. Garden/ 6. Cooking program/ 7. Selection of food or Purchase of food/ 8. Food choice/ 9. Consumption of food/ 10. Improvements in fruit and vegetable consumption/ 11. Food budgeting/ 12. Confidence to perform this food work/ 13. Confidence in cooking/ 14. Frequency of using basic ingredients for the preparation of meals/ 15. Confidence in cooking/ 16. Buying less convenience food/ 17. Preparation of meal and snack/ 18. Increased likelihood to taste and experiment with new food/ 19. Increased awareness of food preparation and Production 20. Health Literacy/ 21. Nutrition Knowledge, Attitudes, Practice/ 22. (literacy or literate).tw. 23. or/1-22 24. (Child* or adolesc* or youth or teenag* or student).tw. 25. Intervent*:tw 26. and/23,24, 25 |
| **EMBASE** | 1. Food literacy/ 2. Nutrition literacy/ 3. Food skills/ 4. Food preparation/ 5. Garden/ 6. Cooking program/ 7. Selection of food or Purchase of food/ 8. Food choice/ 9. Consumption of food/ 10. Improvements in fruit and vegetable consumption/ 11. Food budgeting/ 12. Confidence to perform this food work/ 13. Confidence in cooking/ 14. Frequency of using basic ingredients for the preparation of meals/ 15. Confidence in cooking/ 16. Buying less convenience food/ 17. Preparation of meal and snack/ 18. Increased likelihood to taste and experiment with new food/ 19. Increased awareness of food preparation and Production 20. Health Literacy/ 21. Nutrition Knowledge, Attitudes, Practice/ 22. (literacy or literate).tw. 23. or/1-22 24. (Child* or adolesc* or youth or teenag* or student).tw. 25. Intervent*:tw 26. and/23,24, 25 |
| **ProQuest** | 1. Food literacy 2. Nutrition literacy 3. Food skills 4. Food preparation 5. Garden 6. Cooking program 7. Selection of food or Purchase of food 8. Food choice 9. Consumption of food 10. Improvements in fruit and vegetable consumption 11. Food budgeting 12. Confidence to perform this food work 13. Confidence in cooking 14. Frequency of using basic ingredients for the preparation of meals 15. Confidence in cooking 16. Buying less convenience food in 17. Preparation of meal and snack in 18. Increased likelihood to taste and experiment with new food 19. Increased awareness of food preparation and Production 20. Health Literacy/ 21. Nutrition Knowledge, Attitudes, Practice/ 22. (literacy or literate) in Citation and Abstract 23. or/1-22. in Citation and Abstract 24. randomized controlled trial 25. quasi experimental randomized control trial 26. cluster randomized trial 27. randomized 28. trial 29. groups 30. or/24-29. in Citation and Abstract 31. (Child* or adolesc* or youth or teenag* or student) in Citation and Abstract 32. Intervent* in Citation and Abstract 33. and/23,30, 31, 32 |
| **Web of knowledge/All databases** | 1. Food literacy 2. Nutrition literacy 3. Food skills 4. Food preparation 5. Garden 6. Cooking program 7. Selection of food or Purchase of food 8. Food choice 9. Consumption of food 10. Improvements in fruit and vegetable consumption 11. Food budgeting 12. Confidence to perform this food work 13. Confidence in cooking 14. Frequency of using basic ingredients for the preparation of meals 15. Confidence in cooking 16. Buying less convenience food 17. Preparation of meal and snack 18. Increased likelihood to taste and experiment with new food 19. Increased awareness of food preparation and Production 20. Health Literacy 21. Nutrition Knowledge, Attitudes, Practice 22. (literacy or literate) 23. or/1-22.ti 24. (Child* or adolesc* or youth or teenag* or student).ti 25. and/23,24 26. Intervent*:ti 27. and #23, #24, #25 |
